# An examination into the mental and physical effects of a saffron extract (affron®) in recreationally-active adults: A randomized, double-blind, placebo-controlled study

**DOI:** 10.1080/15502783.2022.2083455

**Published:** 2022-06-07

**Authors:** Adrian L Lopresti, Stephen J Smith

**Affiliations:** a Clinical Research Australia, Perth, Australia; b Murdoch University, College of Science, Health, Engineering and Education, Perth, Australia

**Keywords:** Saffron, exercise, recovery, heart rate, crocus sativus, mood

## Abstract

**Background:**

Saffron, derived from the stigmas of the Crocus Sativus flower, has been shown in several studies to improve mood and wellbeing in adults experiencing low mood and anxiety. The goals of this study were to examine its mental and physical effects in healthy, recreationally active adults.

**Methods:**

In this 6-week, randomized, double-blind, placebo-controlled study, 62 adults engaging in regular exercise were recruited and randomized to receive a placebo or 28 mg daily of a standardized saffron extract (affron®). Self-report outcome measures include the Physical Activity Enjoyment Scale, Profile of Mood States, and Patient-Reported Outcomes Measurement Information System-29. Participants also wore a wrist-worn heart rate, activity, and sleep monitoring device (WHOOP) to measure changes in sleep quality, resting heart rate, and heart rate variability. To help identify mechanisms of action associated with saffron intake, changes in plasma concentrations of brain-derived neurotrophic factor, oxytocin, and neuropeptide Y were also measured.

**Results:**

Based on data collected from all participants, there were no statistically significant between-group differences in changes in any of the outcome measures. However, when changes were analyzed by sex, there were statistically significant greater increases in enjoyment associated with exercise (p =.009) and heart rate variability (p =.001) in male participants taking saffron compared to the placebo. No statistically significant between-group differences were identified in females.

**Conclusions:**

The results of this trial suggest saffron may have beneficial effects in recreationally active males, as evidenced by increased exercise enjoyment and heart rate variability. However, no such benefits were identified in females. Future research using larger sample sizes, varying treatment periods, and additional outcome measures will be required to validate the results from this study and help clarify the mechanisms of action associated with saffron intake.

This study was prospectively registered on 30 October 2020 with the Australian and New Zealand Clinical Trials Registry (Trial ID. ACTRN12621000501842).

## 1. INTRODUCTION

Dietary and herbal supplementation is widely used as a strategy to improve and maintain performance and achieve faster recovery in sports and exercise. Common reasons for dietary supplementation by athletes include the maintenance of good health, the provision of energy and macronutrient needs, performance enhancement, the alleviation of musculoskeletal pain, to support recovery from exercise, and mood enhancement [1,2]. Unfortunately, even though there are countless supplements on the market claiming to enhance physical and mental performance, only a few are supported by robust, evidence-based research [3].

As a mood-enhancing agent, saffron, derived from the stigmas of the *Crocus Sativus* flower, has an increasing body of evidence supporting its efficacy. Based on a meta-analysis of 23 trials, it was demonstrated that saffron at a dose of 28 to 30 mg daily had positive effects on anxiety and depressive symptoms in adults with major depressive disorder and adults experiencing depressive symptoms [4]. At similar doses, saffron has also been shown to have mood-enhancing effects in peri-menopausal women [5], youth experiencing depressive and anxiety symptoms [6], adults with diabetes [7], and adults with metabolic syndrome [8]. It also has demonstrated positive effects on sleep quality in adults experiencing poor sleep [9,10]. However, research into its mood and performance-enhancing impact on athletes is limited. As an adjunct to resistance exercise in untrained young males, saffron supplementation for 6 weeks increased happiness ratings and blood concentrations of dopamine, β-endorphin, and serotonin [11]. In non-active, healthy male university students, larger improvements in muscle strength and reaction time were identified after 10 days of saffron supplementation compared to the placebo [12]. These preliminary studies suggest that as an adjunct to exercise, saffron supplementation may have ergogenic and mood-enhancing effects. However, these studies were conducted on non-active males, and supplementation was delivered at doses much higher than in mood trials (150 to 300 mg daily compared to 28 to 30 mg daily). Due to the high cost of saffron, delivery at these higher doses makes its supplementation cost-prohibitive for most people.

Regular physical activity is associated with improved physical and mental health, reduced rates of morbidity and mortality, and better quality of life [13,14]. However, despite abundant evidence confirming the health benefits associated with exercise, the general adult population spends considerable time being sedentary and little time engaging in physical exercise [15]. Research confirms that feelings of pleasure and enjoyment associated with exercise are positively associated with exercise adherence [16,17]; therefore, increasing exercise enjoyment presents as a means of increasing adherence to exercise. Moreover, strategies to improve recovery after exercise may encourage greater exercise adherence, increase overall pleasure associated with exercise, enhance the mental and physical benefits derived from regular physical activity, reduce the risk of physical injury, and improve overall performance [18–20].

Given the evidence of saffron’s positive effects on mood and sleep in people with mood disturbances, and preliminary evidence of its ergogenic effects, it presents as a possible agent to support mood, motivation, and recovery in physically active adults. Changes in resting heart rate (RHR), heart rate variability (HRV), and sleep quality are indicators of recovery as studies have demonstrated that compared to healthy controls, adults with burnout have an elevated RHR [21] and poorer sleep [22]. Moreover, lower HRV indicates reduced parasympathetic activity [23,24]. In an animal study, saffron administered for 7 days increased HRV and lowered RHR [25]. In a trial on healthy adults with subclinical feelings of low mood and anxiety, saffron attenuated typical stress-induced decreases in HRV during exposure to a laboratory stressor [26]. Several blood markers of chronic stress, fatigue, and mood have been proposed, but inconsistency in findings confirms that there is no perfect biomarker. Brain-derived neurotrophic factor (BDNF) has an important role in neuronal survival and growth [27] and presents as possible maker of chronic stress [28]. In an animal study, rats treated with saffron combined with exercise had significantly greater plasma BDNF concentrations than the exercise only group [29]. Oxytocin is a neuropeptide that interacts closely with neural pathways responsible for processing motivationally relevant stimuli [30], while neuropeptide Y (NPY) influences mood, feeding behavior, sleep regulation, and neuronal growth and remodeling [31]. Oxytocin and NPY have also been proposed as physiological markers of chronic stress [28,32]. The effects of saffron on these neuropeptides have not been previously investigated, although in an animal study, saffron supplementation increased levels of VGF neuropeptide (a polypeptide precursor to several biologically active peptides) in the rat hippocampus [33].

The aims of this randomized, double-blind, placebo-controlled trial were to investigate the effects of chronic saffron supplementation in recreationally active adults on mood, exercise enjoyment and recovery, as measured by changes in RHR, HRV, and sleep patterns. Moreover, potential mechanisms of action associated with saffron supplementation comprising changes in blood concentrations of BDNF, oxytocin, and NPY were investigated. It was hypothesized that compared to the placebo, saffron supplementation would be associated with greater improvements in mood, exercise enjoyment, and recovery; and in the measured stress-related blood markers.

## 2. MATERIALS AND METHODS

### Study design

**Figure 1.**
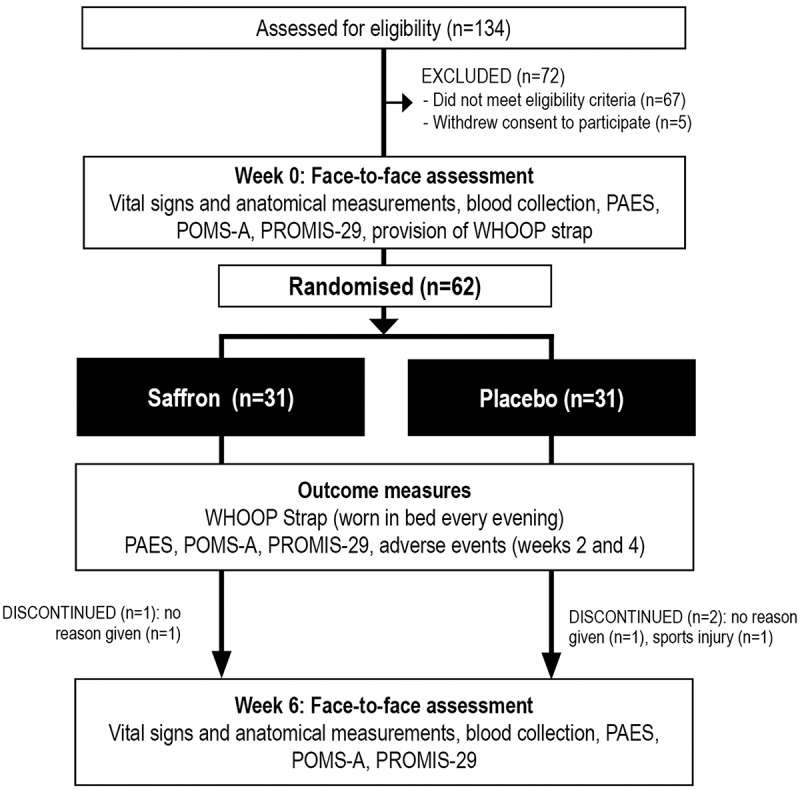
Systematic illustration of study design.

This was a two-arm, parallel-group, 6-week, single-center, randomized, double-blind, placebo-controlled trial (Figure 1). The trial protocol was approved by the Human Research Ethics Committee at the National Institute of Integrative Medicine (approval number 0084E_2021) and all participants gave informed consent. This study was prospectively registered with the Australian and New Zealand Clinical Trials Registry (Trial ID. ACTRN12621000501842).

PAES = Physical Activity Enjoyment Scale, POMS-A = Profile of Mood States, Abbreviated Version, PROMIS-29 = Patient-Reported Outcomes Measurement Information System −29

### Sample size calculation

There have been no previous trials examining the effects of saffron or other herbal extracts on exercise enjoyment in recreationally active adults, so an estimate of effect size on the primary outcome measure could not be reliably undertaken. Therefore, in this exploratory trial, a convenience sample size of 60 adults was planned for recruitment.

### Recruitment and randomization

Social media advertisements and e-mail databases were used to recruit participants between June and August 2021. Interested volunteers visited a website page that provided details about the trial and a link to complete an online screening form that assessed for self-reported exercise frequency, type, duration, and intensity; medication use; history of medical or psychiatric disorders; current injuries or surgeries; alcohol, nicotine, and other drug use; caffeine intake; and supplement and vitamin intake. To assess the severity of depressive and anxiety symptoms, respondents also completed the 4-item Patient Health Questionnaire (PHQ-4). The PHQ-4 has been demonstrated as a reliable and valid brief self-report measure to detect anxiety and depressive disorders in adults [34].

Eligible and consenting participants were randomly allocated to one of two groups (saffron or placebo). To ensure sequence concealment, a randomization calculator was used to create a randomization structure comprising six randomly permuted blocks, containing 10 participants per block. Identification numbers were allocated to participants based on their enrollment order in the study. All tablets were packaged in matching bottles labeled by two intervention codes (held by the sponsor until all data were collected). Study participants and investigators were blind to the treatment group allocation until all outcome data were collected.

### Participants

If evaluated as likely eligible, volunteers participated in a telephone interview where their eligibility was further assessed, and demographic details were obtained. Suitable participants were then required to complete a consent form (online) and attend an in-person assessment approximately 3 to 7 days after the interview. Participant demographic information is provided in Table 1 and eligibility criteria are outlined below.

**Table 1.** Baseline participant details

		Placebo (n = 31)	Saffron (n = 31)	p-value
Age	Mean	41.84	44	.457^a^
	SE	1.9	2.18	
BMI	Mean	24.3	25.39	.131^a^
	SE	0.47	0.53	
Sex	Male	15	22	.070^a^
	Female	16	9	
Marital status	Single	9	11	.587^b^
	Married/ defacto	22	20	
Education	Secondary	17	12	.231^b^
	Tertiary	8	7	
	Post-graduate	6	12	
Systolic blood pressure (mmHg)	Mean	137.93	136.35	.669^a^
	SE	2.44	2.7	
Diastolic blood pressure (mmHg)	Mean	83.32	83.26	.978^a^
	SE	1.5	1.74	
IPAC category	Moderate	17	15	.611^b^
	High	14	16	
IPAC METS	Mean	2220.1	2167.74	.823^a^
	SE	166.62	162.44	
Type of exercise^c^	Aerobic/ fitness classes	16	10	.217^b^
	Running	11	13	
	Cycling	7	8	
	Gym/ resistance exercise	10	12	
	Swimming	2	4	
	Gymnastics	3	1	
	Boxing/ martial arts	1	1	
Self-report questionnaires		n = 31	n = 31	
PAES	Mean	105.35	107.58	.515^a^
	SE	2.62	2.17	
POMS-A: Total mood disturbance	Mean	87.03	89.74	.585^a^
	SE	3.42	3.57	
PROMIS: Mental Health	Mean	47.76	50.08	.192^a^
	SE	1.06	1.4	
PROMIS: Physical Health	Mean	34.79	35.04	.801^a^
	SE	0.7	0.7	
Blood measures		n = 28	n = 28	
BDNF (ng/mL)	Mean	1138.43	1295.43	.194^a^
	SE	81.09	87.76	
NPY (pg/mL)	Mean	13.56	15.16	.526^a^
	SE	1.45	2.04	
Oxytocin (pg/mL)	Mean	2765.93	2960.64	.339^a^
	SE	150.45	134.36	
WHOOP measures		n = 31	n = 30	
HRV	Mean	55.98	50.1	.310^a^
	SE	4.69	3.27	
RHR	Mean	58.55	55.04	.062^a^
	SE	1.24	1.37	
Total sleep time (hours)	Mean	7.1	6.92	.449^a^
	SE	0.12	0.2	
Sleep efficiency (%)	Mean	88.61	89.29	.519^a^
	SE	0.75	0.73	

a = independent samples t-test, b = chi-square analysis, c = many participants engaged in more than one form of exercise

Abbreviations: BDNF – Brain-derived neurotrophic factor; HRV – Heart rate variability; IPAC – International Physical Activity Questionnaire; METS – Metabolic equivalent of tasks; NPY – Neuropeptide Y; PAES – Physical Activity Enjoyment Scale; POMS-A – Profile of Mood States, Abbreviated Version; PROMIS – Patient-Reported Outcomes Measurement Information System; RHR – Resting heart rate

*Inclusion criteria*: Healthy male and female participants aged 18 to 65 years, engaging in nonprofessional, moderate-to-intense aerobic exercise more than 3 times per week, and comprising exercise activities such as cycling, running, swimming, tennis, aerobics, and/or boxing were eligible to participate in this study. Exercise sessions needed to be greater than 45 minutes, participated in for longer than 6 months before the study, and no more than 14 hrs a week. Volunteers were nonsmokers, had a body mass index (BMI) between 18 and 30, reported no plan to start new treatments during the study, and were fluent in English.

*Exclusion criteria*: Participants were considered ineligible if they suffered from medical conditions including, but not limited to: diabetes, hyper- or hypotension, cardiovascular disease, a gastrointestinal disease requiring regular use of medications, gallbladder disease, autoimmune disease, endocrine disease, cancer or malignancy, acute or chronic pain condition, neurodegenerative disease, or neurological disease. Moreover, participants were ineligible if they were diagnosed with a serious psychiatric disorder or scored greater than 8 on the PHQ-4 (indicating moderate-to-severe depression and/or anxiety). Regular use of medications such as anticoagulants, anti-hypertensive drugs, anticholinergics, acetylcholinesterase inhibitors, or steroid medications; or if there was any medication change in the last 3 months or a plan to change medication use during the study also resulted in exclusion from the study. People with significant injuries that may affect their ability to engage in exercise; taking herbal or vitamin, or other nutritional supplements that were reasonably expected to influence study measures; reported a current or 12-month history of illicit drug use; an alcohol intake greater than 14 standard drinks per week; consumed more than 3 cups a day of coffee (or other caffeinated beverage); had any significant surgeries over the last year; or reported any planned major lifestyle change during the study period were also ineligible to participate in the study.

As detailed in Figure 1, from 134 people who completed the initial online screening questionnaire, 67 individuals did not meet the eligibility criteria and 5 individuals withdrew consent to participate in the study. Sixty-two volunteers participated in the study and 59 people completed the study. Details of participant background information and baseline scores of the total recruited sample are detailed in Table 1. Baseline demographic details, questionnaire responses, and blood markers were equivalent in both groups. Three participants withdrew from the study. Reasons for withdrawal included no reason given (n = 2) and sports injury (n = 1).

### Interventions

Saffron and placebo tablets were matched for color coating, shape, and size. The active treatment contained 14 mg of a standardized saffron extract (affron®), derived from the stigmas of *Crocus sativus L*. and standardized to contain >3.5% Lepticrosalides®, a measure of bioactive compounds present in saffron, including safranal and crocin isomers. The saffron stigmas were cultivated in Spain and extracted in the factory of Pharmactive Biotech Products SL (Madrid, Spain) to produce affron®. The tablets were manufactured and packed in an Australian Therapeutic Goods Administration registered plant, and the same excipients (microcrystalline cellulose and calcium hydrogen phosphate) were used in the placebo and saffron tablets. All participants were instructed to continue with their preexisting exercise regimen, and take 1 tablet, twice daily, with or without food, for 6 weeks. Tablet adherence was assessed by the daily use of a mobile phone pill monitoring application, by asking participants every fortnight to provide an estimate of the consistency of tablet intake (0% to 100%), and the return of unused tablets at the final assessment. Treatment blinding was evaluated by asking participants to predict group allocation (saffron, placebo, or unsure) at the end of the study. Participants were asked to continue with their exercise habits and pre and post changes in exercise intensity were assessed by an estimation of metabolic equivalent of tasks (METS) based on the researcher-administered International Physical Activity Questionnaire (IPAC) Short Form [35].

### Outcome measures

*Physical Activity Enjoyment Scale (PAES) (Primary Outcome Measure*): The PAES is a reliable and valid 18-item scale that assesses enjoyment for physical activity [36]. Each question is rated on a 7-point scale with higher scores indicating a greater enjoyment of exercise. The PAES was completed fortnightly.

*Profile of Mood States, Abbreviated Version (POMS-A*): The POMS-A is a psychometrically validated, 40-item, self-report questionnaire that assesses a respondent’s current mood state [37]. Questions are rated on a 4-point scale (not at all to extremely) and scores were calculated for total mood disturbance. The POMS-A was completed fortnightly.

*Patient-Reported Outcomes Measurement Information System −29 (PROMIS-29*): The PROMIS-29 is a validated, health-related quality of life self-report questionnaire [38]. A score was calculated for mental/emotional distress (comprising anxiety and depression subscales) and physical health (comprising pain interference and intensity, and ability to participate in social roles and activities subscales) [39]. The PROMIS-29 was completed fortnightly.

*WHOOP strap*: Each participant wore a WHOOP strap 24 hours a day during the study period. Data collected from the WHOOP during sleep time was used to assess changes in sleep (total sleep time and sleep efficiency), RHR rate during sleep, and HRV during sleep. Compared to polysomnography for 2-stage sleep categorizations (i.e. wake and sleep), the WHOOP exhibits satisfactory sensitivity to sleep (89%) and specificity to wake (95%) [40]. WHOOP quantifies heart rate and HRV [in the form of root mean square of successive differences between normal heartbeats (RMSSD)] via wrist-based photoplethysmogram (PPG) during slow-wave sleep. In a validation study, acceptable agreement was found between the WHOOP strap and electrocardiogram (ECG) derived heart rate and HRV measures [41].

*Brain-derived neurotrophic factor (BDNF*): BDNF is a protein involved in plastic changes related to learning and memory. Changes in BDNF expression occur with pathological aging, several psychiatric diseases, and the regulation of adult brain plasticity [42].

*Neuropeptide Y (NPY)*. NPY is the most abundant peptide found in the mammalian brain [43]. Research from animal and human trials have revealed that NPY has stress-relieving, anxiolytic and neuroprotective properties [32]

*Oxytocin*. Oxytocin is a neuropeptide produced in the hypothalamus. It plays an important role in prosocial behaviors. There is consistent evidence that it is negatively affected by cortisol and is increasingly recognized as an important regulator of human social behaviors, including social decision making, evaluating and responding to social stimuli, mediating social interactions, and forming social memories [44].

Blood samples for plasma concentrations of BDNF, NPY, and oxytocin were collected at baseline and week 6. Blood samples were collected in EDTA tubes in a non-fasted state, although participants were asked to not engage in exercise for 12 hours before their blood collection. Samples were collected throughout the day between the hours of 9am and 6pm. Even though collection time varied between individuals, pre and post-collection times were similar within individuals. EDTA tubes were centrifuged at 1500xg for 15 min and the plasma was stored in a − 80-degree freezer until later analysis. Mature BDNF was measured using the commercially available RayBio human BDNF enzyme-linked immunosorbent assay (ELISA) kit (RayBiotech Life, Peachtree Corners, GA) according to manufacturer instructions. Intra-assay and inter-assay coefficiency of variability (CV%) were <10% and <12% respectively, and the minimum detectable level of human BDNF using this assay kit was 80 pg/ml. Oxytocin was measured using the commercially available Abcam human oxytocin ELISA kit (Abcam, Cambridge, Great Britain) according to manufacturer instructions. Intra-assay and inter-assay CV% were 12.0% and 16.4% respectively, and the minimum detectable level of human oxytocin using this assay kit was 15 pg/ml. NPY was measured using the commercially available RayBio human/mouse/rat NPY ELISA kit (RayBiotech Life, Peactree Corners, GA) according to manufacturer instructions. Intra-assay and inter-assay CV% were <10% and <15% respectively, and the minimum detectable level of human NPY using this assay kit was 0.2 ng/ml.

Intra-assay CVs are calculated by dividing the standard deviation (SD) for each pair of duplicate samples by the duplicate mean then multiplied by 100 (CV% = SD σ/mean μ x 100). Intra-assay CV% is reported as the average of the individual CV%’s. Inter-assay CVs are calculated from low and high controls included in every ELISA plate run. The means and SDs for low and high controls from each plate is calculated and the CV% determined by dividing SDs by the means and multiplying by 100. Inter-assay CV% is reported as the average of the individual CV%’s. CVs of <20% are generally accepted as being good [45].

*Adverse events*: The tolerability of tablet intake was assessed every 14 days via an online question querying adverse effects that were believed to be associated with tablet intake. Participants were also asked to contact researchers if they experienced any adverse effects.

### Statistical analysis

For baseline data, a Pearson’s Chi-square test was used to compare categorical data and an independent samples t-test was used to compare group data for continuous variables. Outcome analyses were undertaken using intention-to-treat (ITT), with all participants retained in originally assigned groups. Generalized Linear Mixed Models (GLMM) are assessed for differences between intervention groups on primary and secondary outcomes over time, with intervention effects assessed by intervention group (placebo and saffron) x time interaction. Time points considered for each measure included: PAES (primary outcome measure) (weeks 0, 2, 4, and 6), POMS total mood disturbance scores (weeks 0, 2, 4, and 6), PROMIS mental health and physical health subscale scores (weeks 0, 2, 4, and 6); WHOOP measures (means of weeks 1 to 6); and blood concentrations for BDNF, NPY, and oxytocin (weeks 0 and 6). Random intercepts were utilized in each model, and covariates age, sex, BMI, and IPAQ METS were included. As an exploratory analysis, separate analyses for PAES scores, WHOOP scores, and BDNF, NPY, and oxytocin concentrations were conducted based on sex (male and female). Where applicable, gamma (with log link function) and normal (with identity link function) target distributions were used. Covariance structures with the best model fit were used to model correlation associated with repeated time measurements in gamma and linear models. Robust estimations were used to handle any violations of model assumptions. Intervention group differences at time points were assessed using simple effects. All data were analyzed using SPSS (version 26; IBM, Armonk, NY). As three separate statistical comparisons were undertaken (i.e. total sample, males, and females), a more conservative critical *p*-value was set at *p* ≤ 0.017 for all analyses (0.05/3).

## 3. RESULTS

### PAES

**Figure 2.**
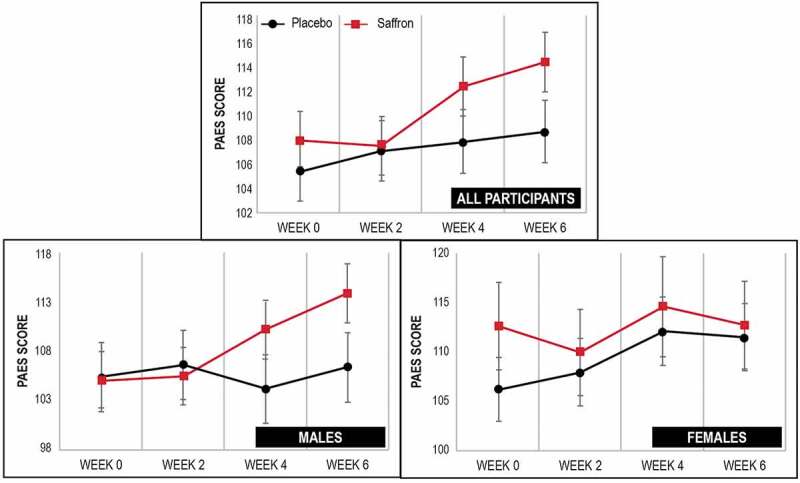
Change in PAES score over time.

As demonstrated in Table 2 and Figure 2, there was a statistically significant increase in PAES scores over time in the saffron group (p = .001) but not the placebo group (p = .098). However, between-group differences were not significantly different (p = .276). An analysis of PAES scores by sex revealed that in males, increases in PAES scores were significantly greater in the saffron group compared to the placebo group over time (p = .009), with a statistically significant increase in PAES scores in the saffron group (p < .001) but not the placebo group (p = .669). In females, changes in PAES scores over time were not significantly different between the placebo and saffron groups (p = .757).

**Table 2.** Change in self-report questionnaires (estimated means)

		Placebo					Saffron					p-value^b^	Effect size
		Week 0	Week 2	Week 4	Week 6	p-value^a^	Week 0	Week 2	Week 4	Week 6	p-value^a^		
ALL PARTICIPANTS (placebo, n = 31; saffron, n = 31)													
PAES	Mean	105.48	107.12	107.89	108.73	.098^†^	107.99	107.57	112.42	114.44	.001^†^	.276	.21
	SE	2.37	2.43	2.45	2.44		2.48	2.5	2.62	2.58			
POMS-A: Total mood disturbance	Mean	86.23	84.41	82.26	76.71	.001^†^	90.42	88.53	89.67	83.08	.014^†^	.787	.10
	SE	3.75	3.72	3.66	3.39		3.93	3.87	3.98	3.63			
PROMIS: Mental health	Mean	47.44	45.7	45.95	45.23	.056	50.35	47.57	48.4	47.16	.009^†^	.922	.11
	SE	1.19	1.28	1.24	1.13		1.26	1.32	1.31	1.17			
PROMIS: Physical health	Mean	34.8	34.15	33.72	32.83	.014^†^	34.98	34.61	34.21	33.14	.021	.989	.02
	SE	0.65	0.65	0.65	0.63		0.65	0.65	0.66	0.62			
PAES	MALES (placebo, n = 15; saffron, n = 22)												
	Mean	105.17	106.45	103.92	106.2	.669	104.87	105.25	110.07	113.81	<.001^†^	.009^††^	.59
	SE	3.57	3.6	3.58	3.62		2.94	2.97	3.04	3.09			
	FEMALES (placebo, n = 16; saffron, n = 9)												
	Mean	106.21	107.97	112.15	111.55	.101	112.67	110.01	114.66	112.78	.979	.757	.28
	SE	3.25	3.42	3.5	3.41		4.47	4.41	5.1	4.47			

^†^statistically significant within-group changes. ^††^statistically significant between-group changes. Results (estimated means) are generated from generalized, mixed-effects models adjusted for BMI, IPAC METS, age, and sex. ^a^P-values are generated from repeated-measures generalized, mixed-effects models adjusted for BMI, IPAC METS, age, and sex (time effects baseline and week 6). ^b^P-values are generated from repeated-measures generalized, mixed-effects models adjusted for BMI, IPAC METS, age, and sex (time x group interaction).

Up and down bars depict standard error bars

### Self-report questionnaires

As demonstrated in Table 2, there were no statistically significant between-group differences in changes in the POMS-A total mood disturbance score (p = .787), or the PROMIS mental health (p = .922) and physical health (p = .989) scores.

### WHOOP measures

As demonstrated in Table 3 there were no statistically significant between-group differences in changes in HRV (p = .502), RHR (p = .289), total sleep time (p = .598), or sleep efficiency (p = .170) over time. Analysis of changes by sex revealed that in males, there was a statistically significantly larger increase in HRV over time in the saffron group compared to the placebo group (p = .001). From baseline to week 6, there was a statistically significant increase in HRV (8.99%) in the saffron group (p = <.001) and a near statistically significant decrease (5.74%) in the placebo group (p = .029). In males, there were no other statistically significant between-group differences in change in WHOOP measures over time. In females, there were no statistically significant between-group differences in change in WHOOP measures over time.

**Table 3.** Change in WHOOP data (estimated means)

		Placebo							Saffron							p-value^b^	Effect size
		Week 1	Week 2	Week 3	Week 4	Week 5	Week 6	p-value^a^	Week 1	Week 2	Week 3	Week 4	Week 5	Week 6	p-value^a^		
ALL PARTICIPANTS (placebo, n = 31; saffron, n = 30)																	
HRV	Mean	47.98	48.88	47.87	47.49	46.89	47.77	.936	49.1	49.2	48.98	48.83	50.59	50.85	.202	.502	.18
	SE	1.15	1.18	1.16	1.16	1.15	1.19		1.19	1.2	1.2	1.19	1.24	1.24			
RHR	Mean	56.63	56.05	56.66	56.9	56.96	56.06	.378	56.63	57.39	56.92	57.39	56.83	57.21	.371	.289	.23
	SE	0.14	0.62	0.53	0.6	0.56	0.64		0.15	0.65	0.54	0.61	0.55	0.63			
Total sleep time	Mean	7.04	6.96	6.90	7.01	6.92	6.98	.645	6.99	6.98	7.16	6.90	7.04	6.83	.183	.598	.11
	SE	0.02	0.10	0.14	0.11	0.13	0.13		0.02	0.10	0.14	0.11	0.13	0.12			
Sleep efficiency (%)	Mean	88.86	88.02	87.83	89.17	89.00	88.85	.978	88.86	89.10	88.75	88.18	89.11	88.53	.475	.170	.09
	SE	0.02	0.42	0.51	0.42	0.47	0.48		0.02	0.44	0.52	0.41	0.46	0.46			
MALES (placebo, n = 15; saffron, n = 22)																	
HRV	Mean	50.87	49.57	49.44	50.12	48.33	47.95	.029	50.29	50.47	51.12	50.61	51.73	54.81	<.001^†^	.001^††^	.97
	SE	1.53	1.49	1.49	1.51	1.48	1.46		1.25	1.27	1.29	1.28	1.31	1.38			
RHR	Mean	54.59	54.98	55.71	55.63	55.73	55.46	.272	54.9	55.65	54.9	55.05	55.03	54.88	.980	.540	.24
	SE	0.18	0.74	0.66	0.64	0.69	0.78		0.15	0.63	0.55	0.53	0.56	0.64			
Total sleep time	Mean	7 .14	6.94	7.00	7.08	7.05	7.01	.495	7.06	7.07	7.12	7.06	7.05	7.05	.937	.843	.11
	SE	0.05	0.14	0.16	0.15	0.18	0.19		0.04	0.12	0.14	0.12	0.15	0.15			
Sleep efficiency (%)	Mean	89.10	88.20	87.81	89.46	89.80	89.11	.986	89.11	89.10	89.12	88.24	89.59	88.45	.196	.260	.19
	SE	0.03	0.56	0.80	0.57	0.67	0.63		0.03	0.48	0.69	0.47	0.55	0.51			
FEMALES (placebo, n = 16; saffron, n = 9)																	
HRV	Mean	44.32	47.28	45.45	44.07	44.65	46.33	.251	47.97	47.82	45.56	46.16	49.41	43.91	.075	.092	.42
	SE	1.82	1.94	1.9	1.85	1.87	1.97		2.64	2.63	2.5	2.54	2.72	2.41			
RHR	Mean	59.63	58.04	58.48	59.09	59.09	57.48	.034	59.26	60.02	60.27	61.54	59.61	61.33	.123	.062	.76
	SE	0.15	1.07	0.83	1.11	0.88	0.99		0.2	1.48	1.11	1.49	1.14	1.33			
Total sleep time	Mean	6.91	6.93	6.78	6.92	6.77	6.92	.940	6.89	6.82	7.30	6.60	7.05	6.37	.007^†^	.140	.59
	SE	0.01	0.14	0.24	0.17	0.20	0.17		0.02	0.18	0.33	0.20	0.27	0.19			
Sleep efficiency (%)	Mean	88.49	87.72	87.72	88.74	88.12	88.45	.953	88.53	89.32	88.10	88.26	88.22	88.96	.652	.783	0.11
	SE	0.03	0.67	0.62	0.65	0.66	0.76		0.04	0.91	0.81	0.83	0.86	0.95			

^†^statistically significant within-group changes. ^††^statistically significant between-group changes. Results (estimated means) are generated from generalized, mixed-effects models adjusted for BMI, IPAC METS, age, sex, and corresponding baseline values. ^a^P-values are generated from repeated-measures generalized, mixed-effects models adjusted for BMI, IPAC METS, age, sex, and corresponding baseline values (time effects baseline and week 6). ^b^P-values are generated from repeated-measures generalized, mixed-effects models adjusted for BMI, IPAC METS, age, sex, and corresponding baseline values (time x group interaction).

### Blood markers

As demonstrated in Table 4 there were no statistically significant between-group differences in changes in BDNF (p = .506), NPY (p = .994), or oxytocin (p = .793) over time. An analysis of changes by sex also revealed there were no statistically significant between-group differences in changes in these blood measures over time.

**Table 4.** Change in blood markers (estimated means)

		Placebo			Saffron			p-value^b^	Effect Size
		Week 0	Week 6	p-value^a^	Week 0	Week 6	p-value^a^		
ALL PARTICIPANTS (placebo, n = 28; saffron, n = 28)									
BDNF (ng/mL)	Mean	1129.16	959.42	.012^†^	1143.13	910.5	<.001^†^	.506	.13
	SE	18.71	64.01		19.54	60.87			
NPY (pg/mL)	Mean	12.68	14.62	.074	12.70	14.66	.072	.994	.00
	SE	0.41	1.00		0.42	1.01			
Oxytocin (pg/mL)	Mean	2766.58	2368.76	.002^†^	2796.72	2441.49	.006^†^	.793	.05
	SE	23.99	121.95		25.14	125.83			
MALES (placebo, n = 14; saffron, n = 20)									
BDNF (ng/mL)	Mean	1234.25	1175.98	.615	1255.71	963.36	.001^†^	.095	.40
	SE	25.64	112.62		21.73	77.17			
NPY (pg/mL)	Mean	11.92	13.47	.234	11.66	13.80	.055	.720	.08
	SE	0.62	1.14		0.51	0.98			
Oxytocin (pg/mL)	Mean	2871.46	2374.00	.008^†^	2917.25	2490.76	.009^†^	.745	.07
	SE	35.98	176.94		30.44	155.30			
FEMALES (placebo, n = 14; saffron, n = 8)									
BDNF (ng/mL)	Mean	1002.28	757.13	.001^†^	992.74	860.63	.189	.339	.29
	SE	22.51	62.72		29.78	94.38			
NPY (pg/mL)	Mean	13.93	16.37	.219	13.78	15.02	.604	.708	.12
	SE	0.42	1.91		0.56	2.32			
Oxytocin (pg/mL)	Mean	2623.69	2323.45	.086	2601.09	2399.46	.390	.737	.10
	SE	30.50	167.59		41.30	229.15			

^†^statistically significant within-group changes. Results (estimated means) are generated from generalized mixed-effects models adjusted for BMI, IPAC METS, age, sex, and corresponding baseline values. ^a^P-values are generated from repeated-measures generalized, mixed-effects models adjusted for BMI, IPAC METS, age, sex, and corresponding baseline values (time effects baseline and week 6). ^b^P-values are generated from repeated-measures generalized, mixed-effects models adjusted for BMI, IPAC METS, age, sex, and corresponding baseline values (time x group interaction).

### Intake of supplements

Tablet bottles with remaining tablets were returned at week 6 and a daily medication monitoring phone application was completed by participants. Based on these details, of the participants who completed the study, only one participant did not take more than 90% of their tablets.

### Efficacy of participant blinding

To assess the effectiveness of condition concealment, participants were asked at the completion of the study to predict condition allocation (i.e. saffron, placebo, or unsure). Group concealment was high as 69% of participants were either unsure or incorrectly guessed treatment allocation.

### Change in exercise intensity

An analysis of IPAQ METS from baseline to week 6 indicated there were no statistically significant changes in physical activity over time in either the placebo (p = .078) or saffron (p = .072) group. Moreover, there were no statistically significant between-group differences in changes in physical activity over time (p = .430).

### Adverse events

There were no reports of any adverse events in 84% (26 out of 31) of participants in the saffron group and 94% (29 out of 31) in the placebo group. Reported adverse effects were of mild severity and there were no treatment drop-outs due to adverse effects associated with tablet intake. In the saffron group, self-reported adverse effects included more vivid dreams (n = 2), increased muscle pain (n = 1), and increased thirst (n = 1). In the placebo group there were reports of increased headaches (n = 1) and sleep disturbances (n = 1). There were no statistically significant between-group differences in changes in BMI (p = .234), systolic (p = .085), or diastolic (p = .183) blood pressure over time.

## 4. DISCUSSION

In this 6-week, randomized, double-blind, placebo-controlled trial, supplementation with 14 mg, twice daily of a saffron extract (affron®) in recreationally active adults was associated with improvements in self-reported exercise enjoyment and mood; however, these improvements were not significantly different to participants taking a placebo. When changes in exercise enjoyment were analyzed by sex, there was a significantly-greater improvement in males taking saffron compared to the placebo, but no between-group differences were observed in females. However, as these findings were only based on data collected from 37 males and 25 females, and there was an uneven group distribution, they should be considered preliminary. There were no significant differences in the activity levels, mean age, or baseline self-report measures between the sexes, making these unlikely variables accounting for the different sex-based findings. Therefore, hormonal differences between the sexes could account for the differing beneficial effects of saffron supplementation in males and females. The mean age of 45 years in females recruited in this trial suggests many females were in the menopausal transition. There is a paucity of human trials examining the effects of saffron on sex hormones such as testosterone and estrogen, although changes in testosterone, estradiol, progesterone, follicle-stimulating hormone, and luteinizing hormone have been identified in animal studies after saffron supplementation [46–49]. In relation to animal studies examining the effects of saffron on testosterone, the intraperitoneal delivery of saffron for 20 days in mice increased serum testosterone concentrations more than the placebo [49], and in a 6-week study, the oral delivery of a saffron extract in conjunction with resistance exercise in male rats increased testosterone concentrations more than resistance exercise alone [50]. In the only human trial examining the effects of saffron on testosterone, six weeks of saffron supplementation (150 mg daily), in addition to resistance training in young men, increased testosterone concentrations more than the control group [51]. Changes in sex hormones were not measured in this current trial, so its relationship to mood, wellbeing, and physiological changes remain unknown. It is important to note that because this was a 6-week trial, pre- and post-measures were collected at differing hormonal times in the normal 28-day hormonal cycle in females which could be another possible reason for non-significant findings in females. Ovarian hormone exposure can influence several mechanisms involved in regulating vascular function at a cellular level as well as functional outcome measures [52].

As a measure of exercise recovery, a wrist-worn sleep, activity, and heart rate tracker (WHOOP) was worn by participants during the study. An examination of changes in HRV, RHR, total sleep time, and sleep efficiency revealed there were no significant differences in changes in these measures between the placebo and saffron groups. However, when changes were examined by sex, males in the saffron group experienced a significantly-greater increase in HRV compared to the placebo group. HRV is a proxy measure of the parasympathetic response, and increases were identified in males supplemented with saffron. Reasons for these sex differences require further investigation, although differences in HRV between men and women have been observed, characterized by a lower HRV in women [53,54]. This suggests that saffron may increase parasympathetic activity in males but not females. As mentioned previously, hormonal differences in sex hormones and the lack of control of hormonal phases in menstruating women could account for these differing sex-based findings. How saffron influences HRV requires further investigation but may be via its influence on hypothalamus-pituitary-adrenal (HPA) axis activity, neurotransmitters activity (e.g. dopamine, serotonin, and noradrenaline), smooth muscle activity, or on the gut microbiome which can also affect vagus nerve activity. In an animal study, saffron reduced cortisol concentrations (suggesting a dampening in HPA-axis activity) [55]; however, no change in cortisol was identified in poor sleepers supplemented with saffron for 4 weeks [9]. In animal studies, saffron administration altered concentrations in, and receptor sensitivity to, serotonin, dopamine, and noradrenaline [56–59]. In a human trial on untrained young males, saffron supplementation for 6 weeks was associated with increased happiness and increases in blood concentrations of dopamine, serotonin, and β-endorphin, although the administered dose of saffron was much higher than used in this study (150 mg compared to 28 mg daily in this study) [11]. It has also been demonstrated in several studies that saffron and its constituents can have a relaxant effect on blood vessels and other smooth muscles, possibly by activating ß2-adrenoceptors, inhibiting histamine H1 and muscarinic receptors and calcium channels, and modulating concentrations of nitric oxide [60]. Moreover, because the gut microbiome can affect vagus nerve activity [61], and saffron can alter gut microbiota [62,63], this presents as another potential mechanism of action associated with saffron intake. Finally, the anti-inflammatory and antioxidant effects of saffron may account for its positive effects on mood and HRV in males [64]. To help confirm reasons for the sex-based differences identified in this study, further research investigating these proposed mechanisms is required

To help understand the potential mechanisms associated with saffron supplementation, changes in plasma concentrations of BDNF, NPY, and oxytocin were examined. However, no between-group differences in change in these hormones were identified. Overall, there were statistically significant reductions in BDNF and oxytocin concentrations over time in both the placebo and saffron groups, and no change in NPY concentrations. However, these findings should be interpreted cautiously as several variables that may influence concentrations of these markers in blood were not controlled for. These include collection times, fasting states, exercise exposure, and in females, variances in the phase of their menstrual cycle. If the overall reduction in BDNF is confirmed in future trials, there is research to indicate that the duration, intensity, and type of exercise can influence BDNF concentrations. For example, in a study on healthy men engaging in different forms of exercise (high‑intensity functional training, high‑intensity interval training, high-intensity power training, and high‑intensity endurance training), increases and decreases in resting BDNF concentrations were seen after exposure to a graded exercise test and the Wingate Anaerobic Test [65]. As participants in this study often engaged in more than one type of exercise, and the intensity and duration of exercise varied significantly, an analysis of biomarker changes by type, intensity, and exercise duration could not be undertaken. It is also important to note that in this study, physically active adults engaging in moderate-to-high activity were recruited and there was no significant change in their physical activity over time. As no change in NPY was observed over time in both the saffron and placebo groups, it may be that NPY concentrations were already at high levels, thereby resulting in ceiling effects. In several animal studies, physical exercise increased NPY concentrations in the blood and brain [66,67]. The influence of saffron in people with suspected low NPY, BDNF, and oxytocin concentrations such as physically inactive people, people experiencing significant stress, adults with mental health problems, and people with other health conditions such as cardiovascular diseases will be important to investigate in future trials [32,44,68,69].

### Limitations and directions for future research

There are several study limitations that may influence the robustness of the findings. As already discussed, the small sample size, particularly after sex-based, sub-group analyses, and the unequal sex distributions reduced the study power, thereby increasing the likelihood of both type 1 (false positive) and type 2 (false negative) error. The results of this study, therefore, require confirmation, utilizing larger sample sizes with a more equal sex distribution. The WHOOP was chosen to measure sleep and heart rate because of its cost-effectiveness, ease of use, and sound reliability and validity [40,41]. However, in future trials, it may be more prudent to utilize gold-standard measures of sleep and HRV such as ECG, electroencephalogram, or actigraphy measures. The non-significant blood biomarker findings should be interpreted cautiously and in future trials it will be prudent to collect morning fasting samples at least 12 hours after any vigorous physical activity. Since the aim of this study was to examine the effects of chronic saffron intake, longer baseline measures obtained from the WHOOP strap (e.g. RHR, HRV, and sleep) were not undertaken. In future trials, it will be useful to conduct baseline assessments for one to two weeks before treatment administration.

Areas of investigation in future trials include examining the effects of saffron delivered over both shorter and longer periods, and at varying doses, particularly as ergogenic effects have been identified in previous studies at doses of 150 to 300 mg daily [11,12]. Examining the effects of saffron as an adjunct to an exercise intervention in people at varying age, fitness levels, mood and physical health, and exercise motivation will also be helpful to understand the potential of saffron to influence mood, exercise satisfaction, recovery, performance, activity levels, and exercise adherence. As the population recruited in this study were healthy and active, floor or ceiling effects on many of outcome measures were likely. Investigating the effect of saffron supplementation based on exercise type, duration, and exercise intensity will also be helpful in the future. This could be achieved by recruiting a more homogeneous population, and/or by prescribing specific aerobic and/or resistance training programs.

In conclusion, the results from this 6-week, randomized, double-blind, placebo-controlled trial revealed that saffron supplementation in recreationally active males, but not females, were associated with increases in self-reported exercise enjoyment and HRV. However, there were no statistically significant between-group differences in changes in sleep, RHR, or plasma concentrations of BDNF, NPY, or oxytocin. Future trials using larger sample sizes, varying treatment periods, and gold-standard outcome measures will be important to confirm findings from this study.
